# First person – Sudhakar Krittika

**DOI:** 10.1242/bio.044966

**Published:** 2019-06-15

## Abstract

First Person is a series of interviews with the first authors of a selection of papers published in Biology Open, helping early-career researchers promote themselves alongside their papers. Sudhakar Krittika is first author on ‘Evidence of diet protein restriction regulating pupation height, development time and lifespan in *Drosophila melanogaster*’, published in BiO. Sudhakar is a PhD student in the lab of Dr Pankaj Yadav at the School of Chemical & Biotechnology, SASTRA Deemed to be University, Tamil Nadu, India, investigating the role of diet restriction on the life-history traits and clock genes of the fruit fly, *Drosophila melanogaster*.


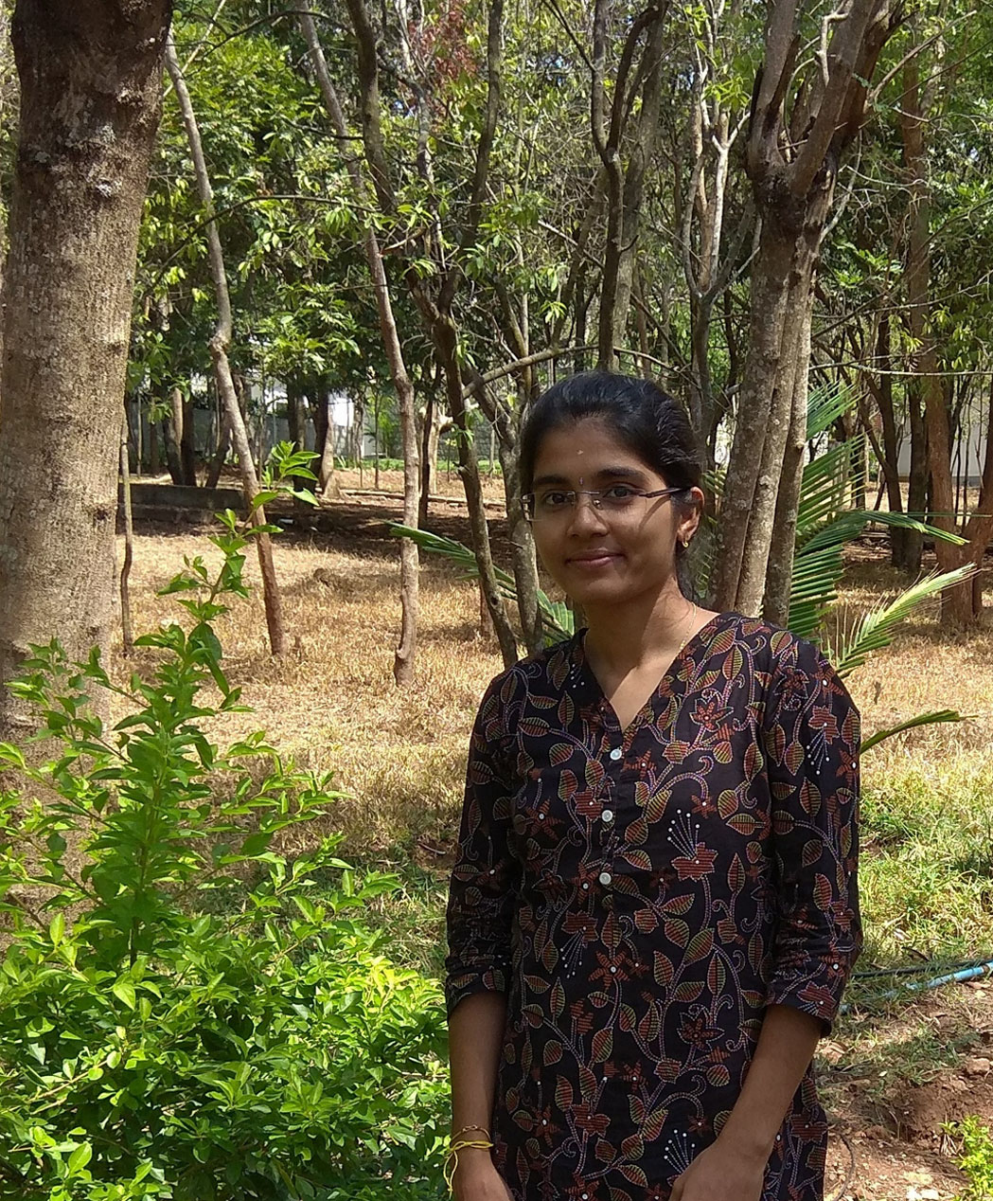


**Sudhakar Krittika**

**What is your scientific background and the general focus of your lab?**

I have obtained my bachelor's and Master's degree in Biochemistry. My first hands-on experience in a fully-fledged research lab was as a part of my Master's degree project, where my focus was Syntaxin 1 protein in ARPE19 cells. Further, for my PhD I joined Dr Pankaj Yadav's Fly lab at SASTRA University as an INSPIRE-Junior Research Fellow, where I was exposed to concepts on the circadian rhythms and diet restriction (DR) in *Drosophila melanogaster*. Our lab focus is aimed towards understanding the role of DR on the fitness and fitness-related traits, activity profile, metabolism and ultimately aging in fruit flies.

**How would you explain the main findings of your paper to non-scientific family and friends?**

Diet restriction is defined as the reduction of certain nutrient/s concentration in the food without causing malnutrition. Fruit flies are small insects commonly found in our kitchens especially around bananas. So using nutrition as a medium to increase lifespan can be the best alternative to running to doctors with physiological ailments. We think ‘eat less, live longer’ is very much suitable for increasing the years of healthy life. In this study, we found that very low protein diet can increase the flies' development time and lifespan, but reduces its feeding rate and the height at which it pupates in the vial. The results also show that the series of restricted protein have positively contributed to the wellbeing of the flies and their lifespan as compared to that of control (unlimited nutrition) food.

**What are the potential implications of these results for your field of research?**

The effect of diet restriction on pupation height is the first of its kind. This will contribute to the current understanding of the preference of pupation site for fruit flies. The debate of correlation between development time and pupation height has been tested and the results show negative correlation between these traits under DR, showing that very low protein reduces pupation height and larval feeding rate, alongside increasing the development time and lifespan of the flies. Hence, the study of energy reserves and storage under diet has been put to the test and therefore will open new challenges for investigation.

**What has surprised you the most while conducting your research?**

The response of flies under the series of imposed diets with so much variation itself was a surprise. A difference of only 10% of yeast among the tested diets resulted in understanding the concentration that can be detrimental and also the concentration that was sufficient to witness more positive effects than the control. The effect of DR on the pupation height and larval feeding rate was a beautiful surprise that I will always cherish, moreover making it a first time report of DR influence on pupation height.

“The effect of DR on the pupation height and larval feeding rate was a beautiful surprise…”

**What, in your opinion, are some of the greatest achievements in your field and how has this influenced your research?**

The first ever reports on diet restriction were no less than a scientific breakthrough. Not many people doubt the nutritional value of unlimited or a rich nutritive food and its implications on health. But thankfully this has led to the concept of minimizing the nutritional intake (DR), yet reaping beneficial effects. In India, there is an old saying that states ‘food itself is medicine’, and decades after this, people have come up with the concept of DR, without which our research would not have been possible.

“In India, there is an old saying that states ‘food itself is medicine’…”

**Figure:**
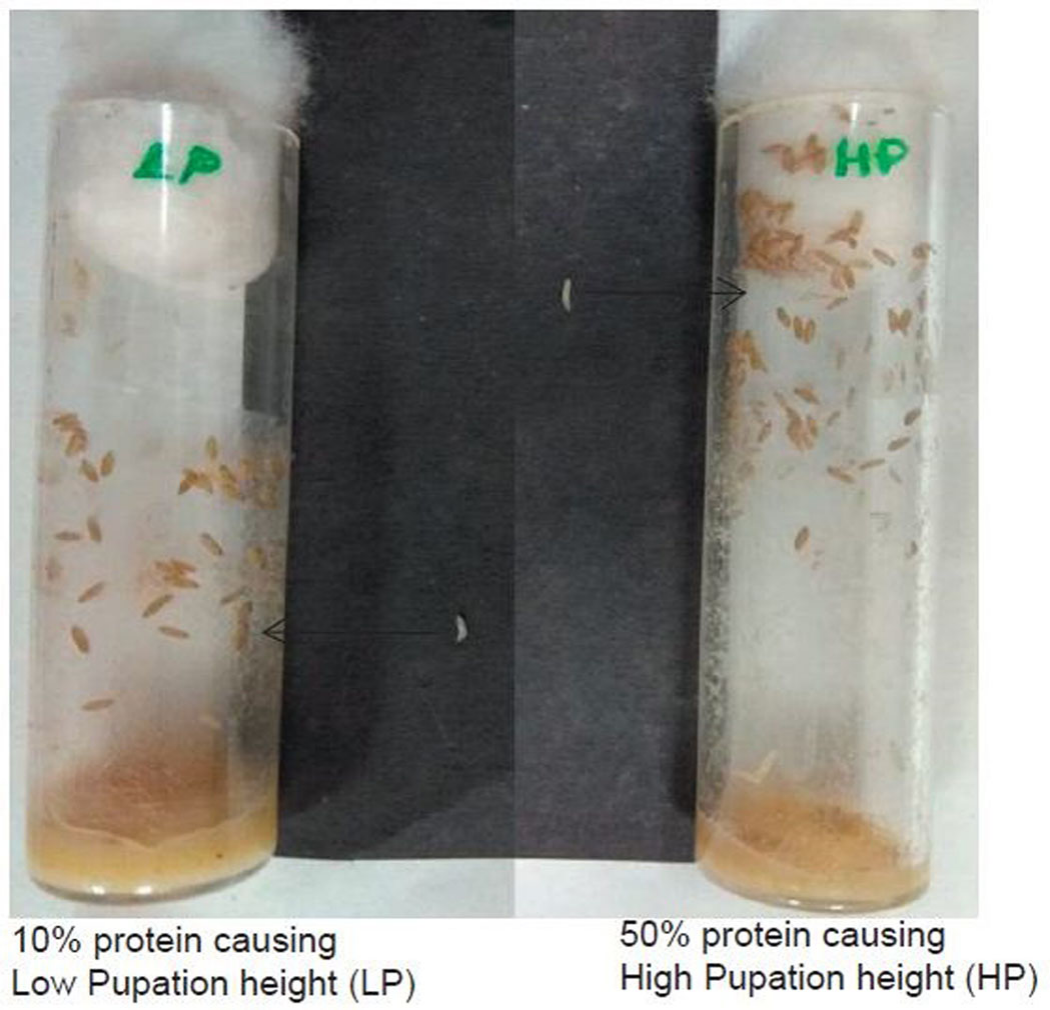
**Dietary protein concentrations of 10% and 50% cause low and high pupation height in *D. melanogaster*, respectively.**

**What changes do you think could improve the professional lives of early-career scientists?**

Recognition for promising research objectives and proposals and timely financial support by the funding agencies could benefit early researchers. There must be multiple funding agencies to which the early-career scientists can apply, so that the ideas don't remain stagnant. It would be beneficial if researchers are given open or institution based free access to research and review articles.

**What's next for you?**

Currently, I am enjoying my flies' response to my research and am hopeful of completing my PhD in two years. I would like to extend my research field to explore more of DR and cancer metabolism and try to improve myself as an independent researcher through post-doctoral training.
